# A Community-Directed Integrated *Strongyloides* Control Program in Queensland, Australia

**DOI:** 10.3390/tropicalmed3020048

**Published:** 2018-05-04

**Authors:** Adrian Miller, Elizebeth L. Young, Valarie Tye, Robert Cody, Melody Muscat, Vicki Saunders, Michelle L. Smith, Jenni A. Judd, Rick Speare

**Affiliations:** 1 Ellengowan Drive, Charles Darwin University, Darwin 0909, Northern Territory, Australia; 2 Woorabinda Multi-Purpose Health Service, Queensland Health, 1 Munns Drive, Woorabinda, QLD 4713, Australia; Elizabeth.Young3@health.qld.gov.au (E.L.Y.); adrian.m@gmail.com (V.T.); Robert.Cody@health.qld.gov.au (R.C.); 3 Aboriginal and Torres Strait Islander Health, Faculty of Science, Health, Education and Engineering, University of the Sunshine Coast, Sippy Downs, QLD 4556, Australia; mmuscat@usc.edu.au; 4 Australian Research Alliance for Children and Youth (ARACY), Griffith Criminology Institute, Brisbane, QLD 4001, Australia; vickisaunders@bigpond.com; 5 School of Health and Exercise Sciences, Faculty of Health and Social Development, University of British Columbia, Kelowna, BC V1Y 1V7, Canada; michelle.smith@ubc.ca; 6 School of Health Medicine and Applied Sciences, Centre of Indigenous Health Equity Research, Central Queensland University, Bundaberg, QLD 4670, Australia; j.judd@cqu.edu.au; 7 College of Public Health, Medical and Veterinary Sciences, James Cook University, Townsville, QLD 4811, Australia; Rick.Speare@jcu.edu.au

**Keywords:** *Strongyloides stercoralis*, aboriginal, indigenous, soil-transmitted helminths, mass drug administration

## Abstract

This paper describes two phases of a community-directed intervention to address strongyloidiasis in the remote Aboriginal community of Woorabinda in central Queensland, Australia. The first phase provides the narrative of a community-driven ‘treat-and-test’ mass drug administration (MDA) intervention that was co-designed by the Community Health Service and the community. The second phase is a description of the re-engagement of the community in order to disseminate the key factors for success in the previous MDA for *Strongyloides stercoralis*, as this information was not shared or captured in the first phase. During the first phase in 2004, there was a high prevalence of strongyloidiasis (12% faecal examination, 30% serology; *n* = 944 community members tested) that resulted in increased morbidity and at least one death in the community. Between 2004–2005, the community worked in partnership with the Community Health Service to implement a *S. stercoralis* control program, where all of the residents were treated with oral ivermectin, and repeat doses were given for those with positive *S. stercoralis* serology. The community also developed their own health promotion campaign using locally-made resources targeting relevant environmental health problems and concerns. Ninety-two percent of the community residents participated in the program, and the prevalence of strongyloidiasis at the time of the ‘treat-and-test’ intervention was 16.6% [95% confidence interval 14.2–19.3]. The cure rate after two doses of ivermectin was 79.8%, based on pre-serology and post-serology tests. The purpose of this paper is to highlight the importance of local Aboriginal leadership and governance and a high level of community involvement in this successful mass drug administration program to address *S. stercoralis*. The commitment required of these leaders was demanding, and involved intense work over a period of several months. Apart from controlling strongyloidiasis, the community also takes pride in having developed and implemented this program. This appears to be the first community-directed *S. stercoralis* control program in Australia, and is an important part of the national story of controlling infectious diseases in Indigenous communities.

## 1. Background and Introduction

### 1.1. Background

This paper documents two historical phases of a community-directed *Strongyloides stercoralis* control program. The first phase reports on a community-directed *S. stercoralis* control program in the Indigenous community of Woorabinda, central Queensland Australia. The second phase documents the need to have local Indigenous leadership and direction for community-wide health interventions, and the importance of the researchers having cultural humility. Cultural humility is defined as a lifelong process of self-reflection and self-critique whereby the individual not only learns about another’s culture, but one starts with an examination of her/his own beliefs and cultural identities [1]. Cultural humility cannot be collapsed into a single workshop; it is commitment and active engagement in a lifelong process ‘that individuals enter on an ongoing basis with patients, communities, colleagues, and with themselves’ [1] (p. 118). This paper is a testament to Rick Speare’s cultural humility across his research career.

Note that this paper is conspicuously published well after the first phase of this project. Following a strongyloidiasis-related fatality, the first phase originally focused on the clinical outcomes of an ivermectin mass drug administration [MDA] intervention to address *S. stercoralis* in the community. However, while it was evident that ivermectin MDA clearly had a significant effect on decreasing the prevalence of *S. stercoralis* among Woorabinda residents, the real success of this intervention lay in the self-determination of the community and the community health team, who drove the health promotion program to address strongyloidiasis in the community. Without this commitment from the community health team, and their relationships and trust with community members, there would have been little chance of the successful implementation of the ivermectin MDA.

This paper forms part of a new study that explores barriers and enablers to addressing infectious diseases in Indigenous Australian communities. The community voiced their concerns regarding participating in this study, as they felt: (i) they received no feedback from the original study relating to the ivermectin MDA in their community, so the community requested some researchers from James Cook University, in particular Rick Speare, to assist them with making sense of the data from the MDA, and (ii) their story regarding the success of the community-driven approach to addressing the issue of *S. stercoralis* received little attention. In their view, this was the most important aspect of the success of this program.

To address this shortcoming and the failure in communication, the results of the MDA were shared. The community felt that the community health team, in partnership with the community’s leadership, was the real success of eradicating *S. stercoralis* in Woorabinda. On the community’s invitation, a new research team travelled to Woorabinda to re-engage with community health team in order to capture and share the story of their successful community-driven approach to address this infectious disease, which impacted their community’s health and well-being.

### 1.2. Introduction

Strongyloidiasis is considered one of the most neglected tropical diseases and is estimated to affect over 100 million people worldwide including Indigenous Australians [2,3]. Most cases are chronic, while acute strongyloidiasis is more common in children [4,5]. Chronic strongyloidiasis increases the risk of unpredictable fatal hyperinfection when patients become immunocompromised, malnourished, or immunosuppressed. Hyperinfection can be caused by the administration of corticosteroids to patients with strongyloidiasis [4,5].

In Australia, strongyloidiasis is highly prevalent in Indigenous rural and remote communities, and its management at the individual and community level is sub-optimal [6,7]. For *S. stercoralis,* a prevalence greater than 5% is considered to be hyperendemic, and a public health intervention is required [4,5]. However, although many Indigenous Australian communities appear to have above 5% prevalence, no community-wide control program has been reported. This paper describes the outcome from a community-based intervention to control strongyloidiasis, which was driven by the Aboriginal community in partnership with the community health team.

### 1.3. Context

Woorabinda is a remote, Aboriginal community that is situated approximately 175 km southwest of Rockhampton in Central Queensland, Australia (24°08′05′ S 149°27′22′ E). Woorabinda is the traditional land of the Wadja and Wadjigal people. In 2005, this small discrete Indigenous community had a population of at least 944, with about 191 western-style houses [8].

In 1996, an initial faecal survey for *S. stercoralis* and review of 130 hospital records indicated an overall prevalence of 5% in the community, with the 5–9-year-old age group having the highest prevalence of infection at 14% [9]. In 2000, the testing of patients presenting to the Woorabinda Multipurpose Health Service showed that at least 12% were positive for *S. stercoralis* on faecal testing, and 30% were positive on serology. In addition, a resident who was referred to a tertiary hospital for treatment of another disease, died from hyperinfection.

The Woorabinda Multipurpose Health Service provided health care to the community through a small hospital run by Queensland Health and an active community health team, the Woorabinda Multipurpose Community Health Team. The majority of the team members were Indigenous Australians with ancestral links to the area, and consisted of an Indigenous nurse, a non-Indigenous nurse, several Aboriginal health workers (AHWs), and local residents employed in various roles.

## 2. Approach

In the original phase, the Woorabinda Multipurpose Community Health Team, in partnership with the community, decided to initiate and lead a program to control *S. stercoralis* by involving the whole Woorabinda community and using a combination of treatment and prevention strategies. The goal of the program was to reduce the prevalence of *S. stercoralis* by at least 75% (from around an estimated 30% prevalence to less than 7.5%), with complete elimination from Woorabinda being the goal. The objectives were to: (i) implement a treatment program using agreed protocols; (ii) increase community participation in strategies related to the prevention of *S. stercoralis*; and (iii) decrease environmental risk factors contributing to *S. stercoralis* infection. The program was managed by a steering committee that had members from the Woorabinda Health Service, Woorabinda Council, and Central Queensland Public Health Unit; the committee also included community representatives, and other people were co-opted as required. An advisory panel with specific technical expertise assisted this steering committee.

A program work plan was co-developed, and is detailed in Table 1 to show as an example. Awareness-raising and education about *S. stercoralis* were the first components of the project, and only after the community was saturated with information did the treatment and testing stage begin. Community-based health promotion is people-centered and collectivist [10]. This program involved multiple stakeholders and worked across the community, as demonstrated by the engagement with school and environmental health officers, the council, and community health service. Facilitation and ownership of the program by the community assists with problem-solving, builds the capacity of the community, and therefore enhances successful and sustainable programs [11].

### 2.1. Community Participation

**Figure 1:**
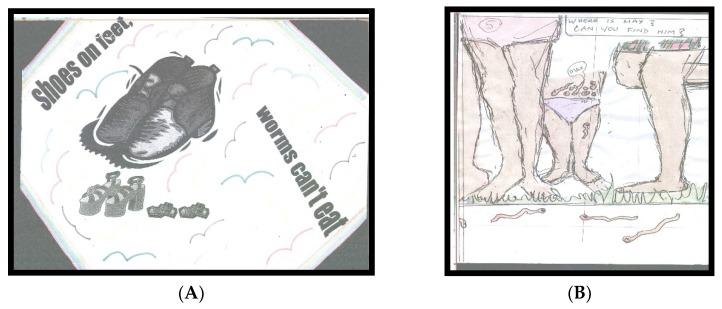

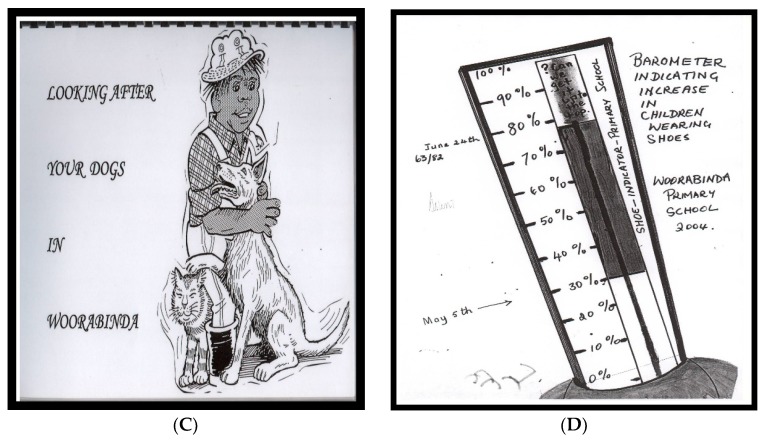
Health promotion material developed during the Woorabinda *Strongyloides* control program. (**A**) Poster on protective footwear; (**B**) Jaime and Aunty June’s comic strip described the life cycle and transmission of *S. stercoralis*; (**C**) Aunty Val’s Gunna Story; (**D**) Shoe barometer.

The Woorabinda Multipurpose Community Health Team worked with stakeholders to develop health education and promotion material to ensure that all of the resources were culturally appropriate and locally relevant. This material focused on explaining *S. stercoralis* and its lifecycle, symptoms of strongyloidiasis, treatment, and prevention of transmission. Female community elders developed several unique health promotion tools, including posters, a comic strip, and Aunty Val’s ‘Gunna Story’ (Figure 1C). In local Aboriginal slang, ‘gunna’ means faeces. An explanation of the local treatment program and prevention strategies encouraged people to play an active role in stopping the transmission of *S. stercoralis*. These health promotion strategies used a multiple intervention model and included: improvement of defects in sanitation systems by house-to-house inspection, personal hygiene, safe disposal of nappies, wearing shoes, and responsible dog ownership. All of these strategies were in line with the principles of the Ottawa Charter [12], which emphasises that multiple strategies across sectors will assist in improving the health of peoples, groups, communities, and populations. A recent study found that wearing shoes decreased the likelihood of infection of schoolchildren with *S. stercoralis* [13].

### 2.2. Involvement of Primary School Children

**Figure 2:**
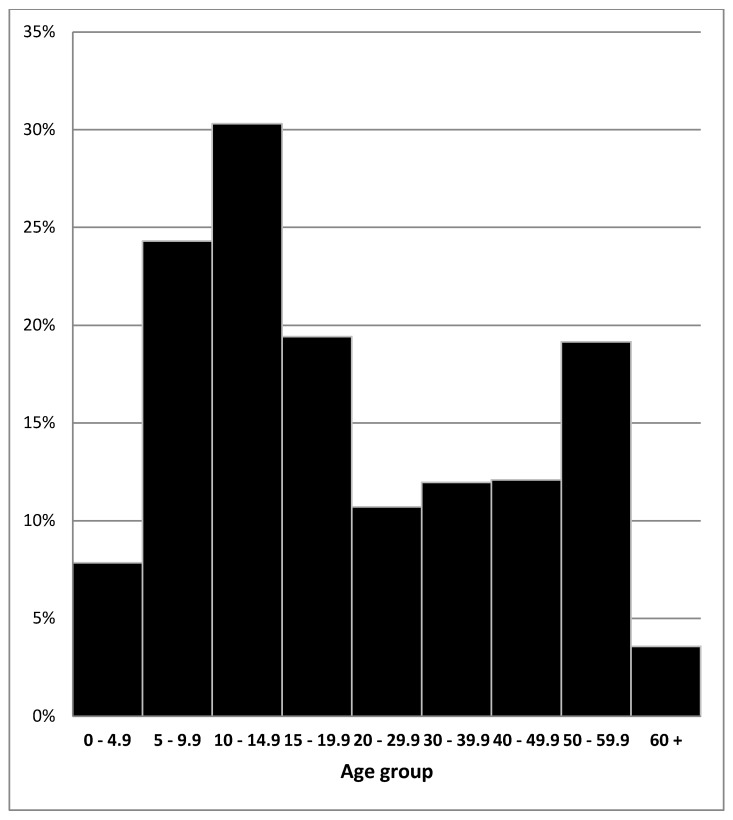
Prevalence of positive *S. stercoralis* serology from an initial survey in phase 1 of the Woorabinda community, July–August 2004.

Teachers and children at the Woorabinda State Primary School were also actively involved. They developed two very specific items: a shoe barometer that measured the percentage of children wearing shoes to classes (Figure 2), and a *Strongyloides* song that was used on the local radio station, particularly preceding announcements about the control program (Table 2). Health promotion programs are most likely to be effective when they are flexible and responsive to local realities [11].

### 2.3. Treat-and-Test

All community residents were urged to participate in a ‘treat-and-test’ survey. Everyone was asked for a sample of blood collected by venipuncture for *S. stercoralis* serology [14], and were treated for *S. stercoralis* at this time. The most effective treatment for *S. stercoralis* is ivermectin [5,15], but ivermectin is not licensed in Australia for treating pregnant women or children with a body weight of less than 15 kg. A less effective alternative option, albendazole, can be used for treatment in the latter category, but was not licensed in Australia for use in pregnant women. The treatment protocol for the Woorabinda program was based on recommendations from the first National Workshop on Strongyloidiasis in September 2001 [16]. This protocol recommended: (1) Children >6 months of age but <10 kg, albendazole: 200 mg daily for three days; (2) Children >6 months of age and 10–15 kg, albendazole: 400 mg daily for three days; and (3) All people except pregnant women >15 kg, ivermectin (Stromectol): 0.2 mg/kg body weight. Women of child-bearing age were offered a pregnancy test prior to treatment with ivermectin. Any person who was subsequently shown to have positive serology for *S. stercoralis* on the initial blood sample was retreated within a month.

### 2.4. Environmental Risk Factors

Environmental health activities were part of the community-wide program. A housing improvement program as part of a state-wide initiative was operating in Woorabinda using Commonwealth and State government funds. A set of priority areas was developed to address environmental risk factors for *S. stercoralis* in the community. Specific actions included repairing malfunctioning toilets and leaking taps and pipes that could cause areas of persistent damp soil.

### 2.5. Serological Results

Over a six-week period, 867 people were treated and tested; the group comprised 46% (404/867) males, and 16.6% (144/867) (95% confidence interval 14.2–19.3) had positive serology. The parasite was present in all age groups, with the highest percentage between 5–20 years, with a peak at 10–15 years and a further rise at 50–59 years (Figure 2). The participation rate was approximately 92% (867/944).

Of the participants who tested positive in July and August 2004, 129 were re-tested in February 2005 after treatment, and 103 had reverted to negative: a cure rate of 79.8%. In February 2005, 140 people who had been initially negative for *S. stercoralis* were re-tested, and none had seroconverted over this six-month period, indicating that transmission at Woorabinda appeared to have ceased.

## 3. Discussion

Most soil transmitted helminth (STH) control programs are driven and managed by health departments, usually from urban centres. Although community-wide control programs for *S. stercoralis* have been advocated since the 1990s [4,17], the only report outside the experimental situation appears to be a Japanese study that monitored and treated a small group of residents on Okinawa, and hence was not a program that engaged the whole community [18]. This Woorabinda program may be the first community-wide program described. It was unusual in that it was initiated, implemented, and managed by the community itself. Woorabinda found the resources—including the funding, person-power, and expertise—that were needed for its successful completion. This was arguably the key factor in achieving a high level of community engagement.

### 3.1. Impact of the Program

The strategy that was adopted was successful in identifying and treating much of the population. The cure rate after two treatments was high, and transmission of *S. stercoralis* at Woorabinda appeared to have ceased. This is the only description of a successful community-led *S. stercoralis* control program. It appears to be the first to use a ‘treat-and-test’ strategy where all of the participants were treated initially for *S. stercoralis*, and subsequent management depended on the test result. Although the long-term effect of the program has not been evaluated, the Woorabinda health centre doctor anecdotally reported to the research team that cases of strongyloidiasis are now rare, and strongyloidiasis is no longer considered a public health issue at Woorabinda. Unfortunately, infection with *S. stercoralis* is not notifiable in Australia; hence, the trend in incidence for Woorabinda is unavailable. Making this parasitic infection notifiable is essential for control and elimination [19].

### 3.2. Local Leadership and Knowledge

The project team used Indigenous and local leadership, with AHW knowledge of the health profile of the local community, the households, and the needs of the community being a very important factor in the success of the mass drug administration program. Additionally, since the AHWs are community members, trust between community members and health staff was high, giving the *S. stercoralis* control program high credibility.

### 3.3. Big Effort, But Worth It

Members of the health team worked very hard during this program, while still performing their usual work tasks. However, they considered that the effort was worth it, since the disease of concern was controlled, and the health team and the community became united on tackling and solving a significant health problem. Over a decade later, yarns with original members of the health team still reflect their sense of pride in their achievement.

### 3.4. Knowledge and Understanding of the Parasite

AHWs and other health staff providing information to community members about *S. stercoralis* facilitated the development of localised knowledge and understanding of the parasite. This was contextualised on the community’s prior knowledge about hookworm, a STH that had been historically present in the community, but had been eliminated. This allowed the team to develop ways to share new knowledge and understanding about *S. stercoralis* and the benefit of the program. Localised communication strategies were developed and delivered in the form of novel health promotion materials such as a children’s song, a children’s story in the local language, localised posters, radio advertisements, and a school-based awareness program, which was exemplified by the shoe barometer. The materials developed were a good illustration that in Aboriginal communities, health promotion materials should be localised and humorised [20].

### 3.5. Development of an Inclusive Governance Model and Skills Development

The team developed a governance model to guide the project, which was a community-driven approach that seconded expert advice at critical points of the program; the steering committee and the advisory panel provided this advice. This approach fostered partnerships between the health and education sectors, researchers, and local government. Strong support for serology tests was provided by Queensland Health laboratory scientists at Rockhampton. AHWs were trained to collect blood (except for infants and babies where phlebotomists were required), and standing orders gave the AHWs authority to administer anthelmintic medications. An implementation protocol with a shared understanding of the commitment required for this program was a core component of the program.

### 3.6. Aspects to Be Improved

A decrease in the number of people affected meant that momentum for the program to progress to elimination was lost. Although a repeat community-wide survey to document the post-program prevalence of strongyloidiasis would have provided valuable information, owing to lack of further targeted resources, no survey was conducted.

## 4. Conclusions

This paper illustrates how a community-directed and led *S. stercoralis* control program using a ‘treat-and-test’ strategy in a remote Australian Aboriginal community brought an outbreak and subsequent high prevalence of strongyloidiasis under control. The use of localised health promotion strategies and materials developed by community members contributed to the program’s success, and set out a clear plan for action for other communities needing to address *S. stercoralis*. Since *S. stercoralis* is usually not a health problem in developed countries and is usually associated with poverty, its high prevalence in this community drew attention to the underlying social determinants of health and the need for social solutions, as well as biomedical interventions.

## Figures and Tables

**Table 1:** Woorabinda *S. stercoralis* Control Program Work Plan.

Months After Commencement	Action	Responsibility *
3–4 months	Formation of a steering committee	DON
	Adoption of program plan	Steering committee
	Appointment of personnel to conduct program	DON
	Develop consent forms: ivermectin, albendazole, beta-HCG (test for pregnancy), release of information.	PO/RN
	Develop data collection tools for treatment—paper and electronic	PO/RN
	Develop drug recording systems	PO/RN
	Develop management flow chart	PO/RN
	Develop education and awareness-raising materials	PO/RN
	Collect baseline data results from previous studies conducted in Woorabinda and develop evaluation measures for comparison: (a) Environmental health and household survey; (b) Animal census.	PO
	Environmental health program commences	EHO, EH Coordinator/HW’s/Council
	Education and awareness-raising commences	HW
6 months	Pilot of initial treatment	DR/RN/HW
	Review and modification of management flow chart	Steering Committee
	Environmental health program continues	EHO, EH Coordinator/HW’s/Council
	Initial serology/treatment of community	DR/RN/HW
	Follow-up of community members not presenting for treatment/re-treatment of positive cases at two weeks	RN/HW
	Education and awareness-raising continues	HW
	Environmental health program continues	EHO, EH Coordinator/HW’s/Council
	Analysis of results from initial treatment and report	RN
12–13 months	First follow-up serology and treatment of resistant/positive cases.	DR/RN/HW
	Follow-up community members who have not presented for serology and treatment/resistant cases	RN/HW
	Analysis of results from first follow-up and report	RN
	Second follow-up treatment for resistant cases	DR/RN/HW
	Follow-up community members not presenting for serology and treatment	RN/HW
	Education and awareness raising continues	HW
	Environmental health program continues	EHO, EH Coordinator/HW’s/Council
	Analysis of results from second follow-up and report	RN
14 months	Third follow-up serology and treatment	DR/RN/HW
	Follow-up community members not presenting for serology and treatment	RN/HW
	Education and awareness raising continues	HW
	Environmental health program continues	EHO, EH Coordinator/HW’s/Council
	Analysis of results from third follow-up and report	RN
21–24 months	Fourth follow-up serology	DR/RN/HW
	Follow-up community members not presenting for serology and treatment	RN/HW
	Education and awareness raising continues	HW
	Environmental health program continues	EHO, EH Coordinator/HW’s/Council
	Analysis of results from fourth follow-up and report	RN
	Refer resistant cases to Dr	RN
	Final report and recommendations	RN
	Second yearly review to confirm eradication	Health Service

* DON—Director of Nursing; PO—Project Officer; EHO—Environmental Health Officer; EH Coordinator—Environmental Health Coordinator; DR—Doctor; Council—Local Shire Council; RN—Registered Nurse; HW—Health Worker.

**Table 2:** The *Strongyloides* Song Developed and Sung by the Woorabinda State Primary Schoolchildren.

The Strongyloides Song
*Words by June Barkworth and adapted by the Woorabinda Schoolchildren. The children and staff of the Woorabinda State School wrote the music. This song was used as a signature tune to herald radio updates on the Strongyloides project.* No, no, no, ‘Mr Worm’ we don’t want you Travelling through our skin and making us sick, With boots and shoes on our feet (stamp, stamp) Blankets on the ground, We’re gon’na stop you from moving around ‘Our bodies’ Yes, yes, yes, ‘Mr Worm’ you have got to go ho, ho, ho, ho, Go, go, go ‘Mr Worm’ we’re getting tough You have no place to live in us. We’re gon’na thrash you out, we’re gon’na move you on …… then Woorabinda will say that ‘Mr Strongyloides’ worm is gone. Yes, yes, yes ‘Mr Worm’ you have got to go. Ho, ho, ho, ho, ho.

## References

[1] Tervalon M., Murray-Garcia J. (1998). Cultural humility versus cultural competence: A critical distinction in defining physical training outcomes in multicultural education. J. Health Care Poor Underser..

[2] Olsen A., van Lieshout L., Marti H., Polderman T., Polman K., Steinmann P., Stothard R., Thybo S., Verweij J.J., Magnussen P. (2009). Strongyloidiasis—The most neglected of the neglected tropical diseases?. Trans. R. Soc. Trop. Med. Hyg..

[3] Puthiyakunnon S., Boddu S., Li Y., Zhou X., Wang C., Li J., Chen X. (2014). Strongyloidiasis—An insight into its global prevalence and management. PLoS Negl. Trop. Dis..

[4] Shield J.M., Page W. (2008). Effective diagnostic tests and anthelmintic treatment for *Strongyloides stercoralis* make community control feasible. PNG Med. J..

[5] Page W., Shield J., O’Donahoo F., Miller A., Judd J., Speare R., Loukas A. (2016). Strongyloidiasis in Oceania. Neglected Tropical Diseases—Oceania.

[6] Miller A., Smith M.L., Judd J.A., Speare R. (2014). *Strongyloides stercoralis*: Systematic review of barriers to controlling strongyloidiasis for Australian Indigenous communities. PLoS Negl. Trop. Dis..

[7] Mounsey K., Kearns T., Rampton M., Llewellyn S., King M., Holt D., Currie B.J., Andrews R., Nutman T., McCarthy J. (2014). Use of dried blood spots to define antibody response to the *Strongyloides stercoralis* recombinant antigen NIE. Acta Trop..

[8] National Regional Profile: Woorabinda Local Government Area. http://www.abs.gov.au/AUSSTATS/abs@nrp.nsf/Previousproducts/LGA37550Population/People12005-2009?opendocument&tabname=Summary&prodno=LGA37550&issue=2005-2009&num=&view=.

[9] Australian Centre for International and Tropical Health and Nutrition (ACITHN) (1996). Environmental Health in Woorabinda: Investigation of Parasite Infections in the Community.

[10] Raeburn J. (1998). and Rootman, I. People Centered Health Promotion.

[11] Judd J., Frankish CJ and Moulton G. (2001). Setting Standards in the evaluation of community-based health promotion programs—A unifying approach. Health Promot. Int. J..

[12] World Health Organization (WHO) (1986). Ottawa Charter for Health Promotion. Health Promot..

[13] Khieu V., Hattendorf J., Schär F., Marti H., Char M.C., Muth S., Odermatt P. (2014). *Strongyloides stercoralis* infection and re-infection in a cohort of children in Cambodia. Parasitol. Int..

[14] Sampson I.A., and Grove D.I. (1987). Strongyloidiasis is endemic in another Australian population group: Indochinese immigrants. Med. J. Aust..

[15] Biggs B.A., Caruana S., Mihrshahi S., Jolley D., Leydon J., Chea L., Nuon S. (2009). Management of chronic strongyloidiasis in immigrants and refugees: Is serologic testing useful?. Am. J. Trop. Med. Hyg..

[16] Page W., Speare R. (2003). Recommendations from the First National Workshop on Strongyloidiasis. http://www.tropicalhealthsolutions.com/sites/default/files//uploaded/Recommendations-1NWS.pdf.

[17] Conway D.J., Lindo J.F., Robinson R.D., Bundy D.A.P. (1995). Towards effective control of *Strongyloides stercoralis*. Parasitol. Today.

[18] Toma H., Shimabukura I., Kobayashi J., Tasaki T., Takara M., Sato Y. (2000). Community control studies on *Strongyloides* infection in a model island of Okinawa, Japan. Southeast Asian J. Trop. Med. Public Health.

[19] Speare R., Miller A., Page W. (2015). Strongyloidiasis: A case for notification in Australia?. Med. J. Aust..

[20] Massey P.D., Miller A., Saggers S., Durrheim D.N., Speare R., Taylor K., Pearce G., Odo T., Broome J., Judd J. (2011). Australian Aboriginal and Torres Strait Islander communities and the development of pandemic influenza containment strategies: Community voices and community control. Health Policy.

